# Aggregation of Gold Nanoparticles in Presence of the Thermoresponsive Cationic Diblock Copolymer PNIPAAM_48_-b-PAMPTMA_6_

**DOI:** 10.3390/polym13234066

**Published:** 2021-11-23

**Authors:** David Herrera Robalino, María del Mar Durán del Amor, Carmen María Almagro Gómez, José Ginés Hernández Cifre

**Affiliations:** 1 Departamento de Química Física, Facultad de Química, Universidad de Murcia, 30100 Murcia, Spain; david.herrerar@um.es (D.H.R.); carmenmaria.almagro@um.es (C.M.A.G.); 2 Departamento de Ingeniería Química, Facultad de Química, Universidad de Murcia, 30100 Murcia, Spain; mariamar.duran@um.es

**Keywords:** gold nanoparticles, thermoresponsive copolymers, dynamic light scattering, surface plasmon resonance

## Abstract

The adsorption of the thermoresponsive positively charged copolymer poly(*N*-isopropylacrylamide)-block-poly(3-acrylamidopropyl)trimethylammonium chloride, PNIPAAM_48_-b-PAMPTMA_6_(+), onto negatively charged gold nanoparticles can provide stability to the nanoparticles and make the emerging structure tunable by temperature. In this work, we characterize the nanocomposite formed by gold nanoparticles and copolymer chains and study the influence of the copolymer on the expected aggregation process that undergoes those nanoparticles at high ionic strength. We also determine the lower critical solution temperature (LCST) of the copolymer (around 42 °C) and evaluate the influence of the temperature on the nanocomposite. For those purposes, we use dynamic light scattering, UV-vis spectroscopy and transmission electron microscopy. At the working PNIPAAM_48_-b-PAMPTMA_6_(+) concentration, we observe the existence of copolymer structures that trap the gold nanoparticles and avoid the formation of nanoparticles aggregates. Finally, we discuss how these structures can be useful in catalysis and nanoparticles recovery.

## 1. Introduction

Gold nanoparticles (AuNPs) with their unique functional properties and easy synthesis have attracted much attention and promoted a variety of applications in biomedicine [1], catalysis [2], colorimetric sensing [3], environmental remediation [4], etc. Since bare nanoparticles in solution tend to aggregate, they are coated with small charged molecules which act as stabilizing agents via electrostatic repulsion. However, if the medium has sufficiently high ionic strength, the electrostatic interaction is screened and aggregation occurs [5] which is usually an unwanted effect. A solution is to add some polymer that is adsorbed onto the nanoparticle surface and gives rise to a core-shell nanoparticle [6,7] or forms some type of network that protects the nanoparticle [8]. 

For such a purpose, an interesting type of polymer is the temperature-sensitive poly(*N*-isopropylacrylamide), or PNIPAAM, because its structural features can be tuned by temperature [9]. It is well-known that high-molecular-weight PNIPAAM dissolved in water undergoes coil-to-globule transition when the temperature exceeds its lower critical solution temperature, LCST ≈ 32 °C [10]. For low-molecular-weight PNIPAAM, the value of the LCST depends on both the length of the chain and the polymer concentration [11]. On the other hand, block copolymers formed by a block of PNIPAAM and a block of cationic nature can easily interact with the negatively charged surface of citrate stabilized AuNPs [12]. An interesting cationic polymer to be used as a cationic block is poly(3-acrylamidopropyl)trimethylammonium chloride or PAMPTMA(+) [13,14]. In this work, we use a variant of the thermoresponsive amphiphilic diblock copolymer poly(*N*-isopropylacrylamide)-block-poly(3-acrylamidopropyl)trimethylammonium chloride formed by 48 NIPAAM monomers and 6 AMPTMA(+) monomers, abbreviated PNIPAAM_48_-b-PAMPTMA_6_(+), as coating agent for AuNPs. The short cationic block allows for an efficient interaction with the AuNPs without masking the PNIPAAM block features. 

The main goal of the present work, which extends previous results by the authors [15], is to elucidate how AuNPs interact with PNIPAAM_48_-b-PAMPTMA_6_(+) and evaluate the ability of this copolymer to avoid AuNPs aggregation when the ionic strength of the medium is high, thus expanding the range of application of AuNPs under unfavorable conditions. Besides, since PNIPAAM_48_-b-PAMPTMA_6_(+) has a thermoresponsive nature, we determine the influence of the ionic strength and the temperature on the different systems: AuNPs, copolymer, and nanocomposite formed after mixing the AuNPs suspension and the PNIPAAM_48_-b-PAMPTMA_6_(+) solution. For that purpose, we use dynamic light scattering (DLS), UV-visible spectroscopy, and transmission electron microscopy (TEM) techniques. We conclude that the nanocomposite prevents AuNPs aggregation and discuss its utility in catalysis and in nanoparticles recovery.

## 2. Materials and Methods

Gold nanoparticles (AuNPs) coated by a negatively charged citrate layer were obtained from Sigma-Aldrich (Saint Louis, MO, USA) in form of a suspension in water with 6 × 10^12^ particles/cm^3^. We checked by DLS that the hydrodynamic diameter was d = 20 nm.

**Figure 1:**
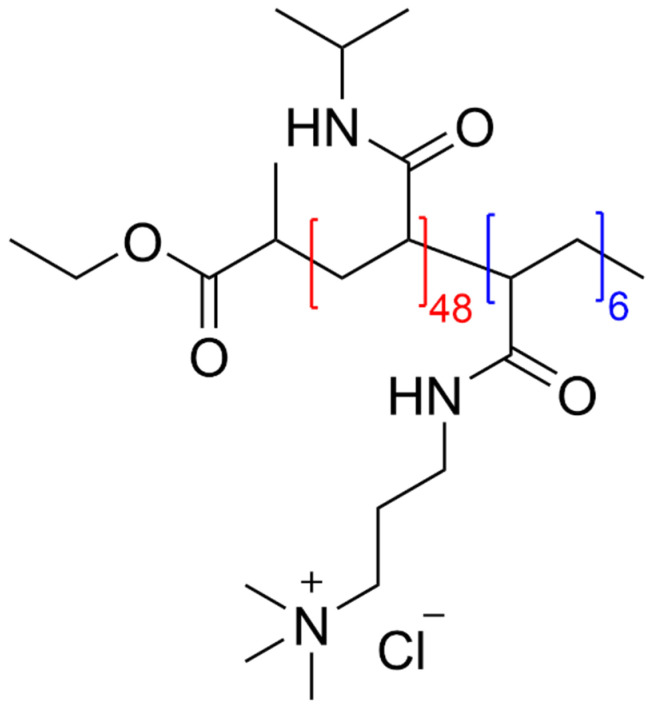
Chemical structure of PNIPAAM_48_-b-PAMPTA_6_(+).

The positively charged diblock copolymer PNIPAAM_48_-b-PAMPTMA_6_(+), abbreviated further from now on as Cop-48/6, was synthesized and kindly supplied by the group of Prof. Nyström of the Department of Chemistry at the University of Oslo, Norway. Details on the synthesis, which is based on the atom-transfer radical polymerization (ATRP) method, can be found in their papers [11,13,16]. The synthesis yields a highly monodisperse copolymer whose molecular weight, determined by the asymmetric-flow field-flow fractionation technique (AFFFF) [13], was M = 8190 g/mol. The chemical structure of this cationic diblock copolymer is displayed in Figure 1.

An aqueous solution of this copolymer of concentration 0.1% *w*/*w* was prepared to be added to the AuNPs suspension. That concentration is appropriate to avoid AuNPs aggregation as shown below (smaller concentrations were not able to prevent aggregation according to preliminary tests).

In order to vary the ionic strength of the medium, I, we used sodium chloride (NaCl). Although in a strict sense the ionic strength of the medium also depends on the Cop-48/6 concentration which is a polyelectrolyte, in practice, we will identify the ionic strength with the NaCl concentration. NaCl with 99% purity was purchased from Panreac (Barcelona, Spain).

### 2.1. Dynamic Light Scattering (DLS): Hydrodynamic Size

The hydrodynamic size was measured by dynamic light scattering (DLS) using a Malvern Zetasizer Nano ZS (Malvern Instruments Ltd., Malvern, UK). That apparatus is equipped with a 4 mW He/Ne laser emitting at 633 nm. We measured the scattering intensity at a 173° angle relative to the source (backscattering). Then, the Malvern Zetasizer software calculates the size distribution function, i.e., the hydrodynamic diameter distribution, from the time autocorrelation function of the scattering intensity fluctuations. Each measurement was set to 15 runs of 15 s each so that the resulting distribution was obtained averaging those of each run. We have checked that this protocol gives reproducible results for the three types of size distributions supplied by the Zetasizer software, namely intensity, volume, and number.

We remind that the distribution by intensity is the primary size distribution obtained from the analysis of the time autocorrelation function mentioned previously and emphasizes the species with the largest scattering intensity, i.e., the largest particles, while the volume and number distributions are derived from the distribution by intensity and stress the species with the highest number of particles.

Caution must be taken in the interpretation of distributions by volume and number because they are obtained assuming the sphericity and homogeneity of particles. In addition, precise knowledge of the actual refractive index of the particles is needed (although the influence of this factor is very small for our system). Finally, it should be taken into account that transformation of the first order intensity distribution to either a volume or number distribution assumes the absence of any error in the intensity distribution. Thus, the distribution by intensity must be that considered in order to characterize particle size whereas distributions by volume and number are adequate to estimate relative populations of the particles and are useful when more than one population is present. In this work, we use the distribution by volume for this purpose.

### 2.2. UV-Vis Spectroscopy: Surface Plasmon Resonance

Absorption spectra were collected by using a T92 + UV-visible spectrophotometer (PG Instruments, Lutterworth, UK). The scanning wavelength range was set from 400 nm to 850 nm being the scanning interval 1 nm. We have checked that the chosen range ensures that the absorption peak in the nanocomposite formed by AuNPs and Cop-48/6 is only due to the metallic nanoparticles. Thus, the surface plasmon resonance (SPR) exhibited by AuNPs is displayed as a strong absorption band in the visible region. The value of the wavelength for this absorption band or plasmon peak depends on the size of the nanoparticle and thus it can be used to detect aggregation of AuNPs. Absorbance measurements were carried out at the lab temperature (25 ± 3) °C because we could not fix an exact temperature value with the instrument. This variation range is not relevant for the conclusions and we will consider that all absorbance measurements were performed at 25 °C.

### 2.3. Transmision Electron Microscopy (TEM)

Transmission electron microscopy (TEM) analysis was carried out with a Jeol 1011 transmission electron microscope (JEOL, Tokyo, Japan) operated at an accelerating voltage of 195 kV, using a Gatan Bioscan Camera model 792 (Gatan, Pleasanton, CA, USA) for imaging acquisition. Samples were prepared by dropcasting (TEM-FCF200CU5, Sigma-Aldrich, Saint Louis, MO, USA) 10 μL of the nanoparticle solution on a 200 mesh Formvar/carbon coated copper/nickel grid and allowing it to dry at room temperature. Excess film was removed with absorbent paper.

## 3. Results

In order to better understand the behavior of the nanocomposite (AuNPs + Cop-48/6) formed by mixing the AuNPs suspension and the copolymer solution, we will initially show the behavior of the AuNPs suspension and the copolymer Cop-48/6 solution independently. For every system, we will characterize its behavior in varying the ionic strength by adding NaCl. In addition, since Cop-48/6 is thermoresponsive, the influence of the temperature on its behavior will be studied and an estimate of its LCST will be given.

### 3.1. The AuNPs Suspension in Varying Ionic Strength

In increasing the ionic strength, the negatively charged surface of the citrate-coated AuNPs is screened by the salt counter-ions and the AuNPs aggregate [5]. In order to characterize such a process, we used the DLS and the UV-visible techniques. Measurements were carried out at 25 °C (AuNPs size does not depend on temperature [12]) and 24 h after adding the salt.

**Figure 2:**
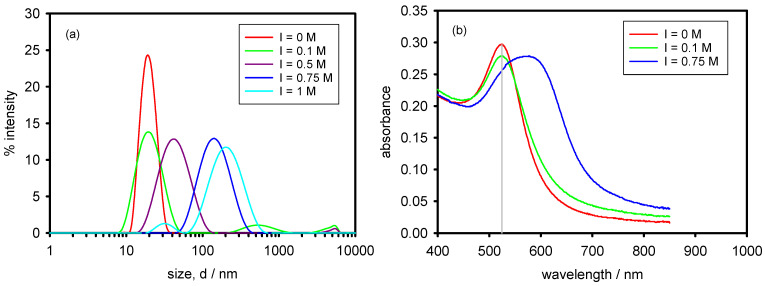
(**a**) Size distributions by intensity of AuNPs in varying NaCl concentration. (**b**) UV-vis spectra of AuNPs in varying NaCl concentration. Measurements were performed 24 h after salt addition and at 25 °C.

Figure 2a (update of Figure 2 in [15]) illustrates the evolution of the size distribution function by intensity (similar to distribution by volume for monodisperse samples) with increasing the ionic strength from 0 M to 1 M. As observed, in between 0 M and 0.1 M, AuNPs size remains almost unaltered. At 0.5 M, the distribution shifts to slightly higher size and above 0.5 M size values over 100 nm are found indicating strong aggregation. From this study, I = 0.75 M was chosen as a worst case to test the ability of Cop-48/6 to prevent AuNPs aggregation. The reason is that value provokes strong aggregation but aggregates remain in suspension for enough time to carry out the measurements.

Figure 2b (update of Figure 3 in [15]) shows the corresponding UV-visible spectra which corroborate the DLS measurements. Thus, at ionic strengths in between 0 M and 0.1 M the wavelength corresponding to the plasmon peak keeps practically around 520 nm (see solid grey line) which is characteristic of AuNPs around 20 nm in hydrodynamic diameter [5,8]. This indicates that aggregation has not appreciably occurred. On the other hand, when the ionic strength is 0.75 M the plasmon peak is clearly shifted to a higher wavelength (phenomenon known as redshift) which indicates that large aggregates were formed. The peak also broads because of the polydispersity of the aggregates.

### 3.2. The Cop-48/6 Solution in Varying Temperature and Ionic Strength

In order to study the behavior of Cop-48/6 in varying temperatures, we used a copolymer solution of 0.01% *w*/*w*. At higher concentrations, copolymer chains strongly aggregate and the LCST cannot be clearly determined. The temperature was varied from 25 °C to 60 °C at intervals of 5 °C. The equilibration time at each temperature was 10 min. From a preliminary study (see Figure S1 in the Supplementary Materials), we checked that time is sufficient for the copolymer aggregates to reach a stable size.

On the other hand, in order to check the influence of the ionic strength on Cop-48/6, we set I = 0.05 M. We used that value because copolymer chains strongly aggregate at higher ionic strength and a value of the LCST cannot be clearly obtained. 

**Figure 3:**
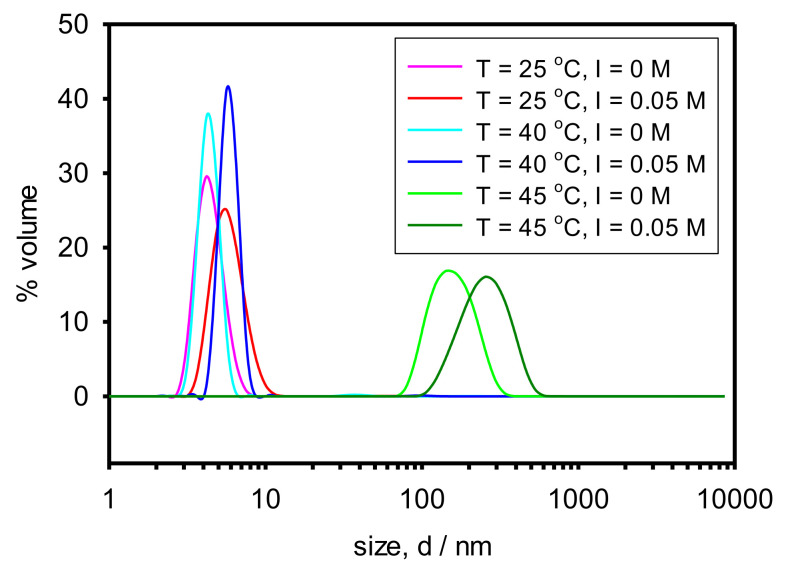
Size distribution by volume of Cop-48/6 for varying temperature and ionic strength.

Figure 3 shows the size distribution by volume for some selected temperatures both with and without added salt. Distribution by volume is used instead of distribution by intensity for systems where the copolymer is present (copolymer solution and nanocomposite) because the distribution by intensity presents several peaks (see Figure S2 in the Supplementary Materials) which are of little relevance (as revealed by distribution by volume) but make the plots unclear. 

Concerning the influence of temperature, it can be appreciated that in between 25 °C and 40 °C a peak between 3 nm and 5 nm is the relevant one. That is approximately the size expected for the copolymer chains assuming they present a random coil conformation and their hydrodynamic radius is similar to the radius of gyration. The latter can be estimated by using the relationship for flexible vinyl polymers Rg = (C_∞_ × n_c–c_ × d^2^_c–c_/6)^1/2^ where C_∞_ is the characteristic ratio, n_c–c_ is the number of the backbone carbon-carbon bonds and d_c-c_ is the carbon-carbon bond length [17]. Assuming C_∞_ = 10.6 for PNIPAAM (the main Cop-48/6 block) [18], n_c–c_ = 54 (48 PNIPAAM monomers + 6 PAMPTMA monomers), and d_c–c_ = 0.154 nm (typical length of the C–C bond), the radius of gyration turns to be Rg = 1.5 nm which implies a diameter of 3 nm. Therefore, most of the copolymer chains are random coils below 40 °C which indicates that the temperature range is below the LCST. However, for 45 °C peaks in between 100 nm and 1000 nm are the relevant ones that clearly correspond to aggregates or networks of copolymer chains indicating that temperature is above the LCST. At the LCST, the copolymer becomes hydrophobic due to the PNIPAAM block (which is much larger than the hydrophilic PAMPTMA(+) block) and different chains aggregate due to hydrophobic interactions. 

Concerning the influence of the ionic strength, it can be appreciated that the slight increase in the ionic strength provokes a slight increase in the size of the aggregates formed above the LCST. It is known that the increase in ionic strength favors the association of the PNIPAAM_8_-b-PAMPTMA_m_(+) chains and can even diminish the LCST [19]. 

**Figure 4:**
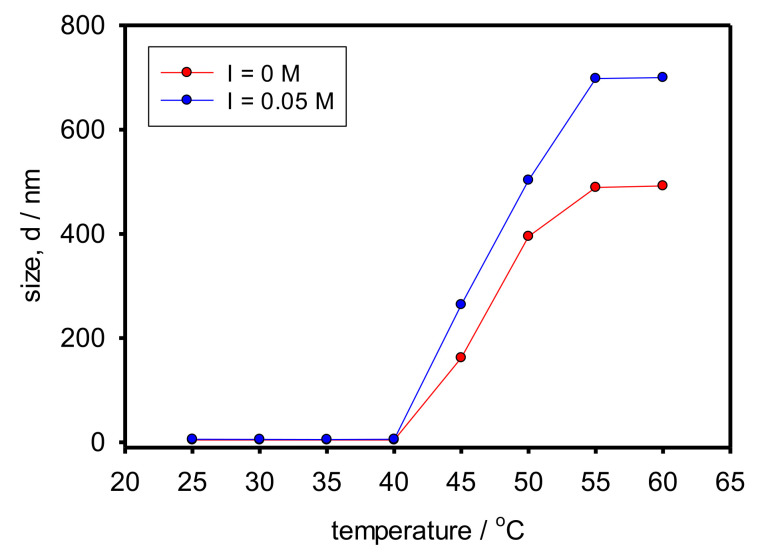
Evolution of the hydrodynamic diameter of Cop-48/6 with the temperature both with and without added salt.

Figure 4 shows the evolution of the copolymer hydrodynamic diameter with the temperature both at I = 0 M and I = 0.05 M. In order to assign a value to the hydrodynamic diameter, we used the peak value corresponding to the most relevant population as revealed by the distribution by volume. 

As observed, independently of the ionic strength, there is a sudden increase of the size between 40 °C and 45 °C. Therefore, we can establish the LCST in that range and estimate its value around 42 °C. As mentioned in the Introduction, the LCST of high-molecular-weight PNIPAAM is 32 °C. A higher value is expected for Cop-48/6 because of the hydrophilic PAMPTMA(+) block and the relatively short PNIPAAM block. It is known that the ratio of lengths of the hydrophobic to the hydrophilic blocks influences the interchain association because of the protective role of the hydrophilic block [19,20]. On the other hand, a steeper size increment and larger aggregates are observed in presence of salt because the screening of copolymer charges favors interchain association [19].

### 3.3. The Nanocomposite (AuNPs + Cop-48/6) in Varying Ionic Strength

**Figure 5:**
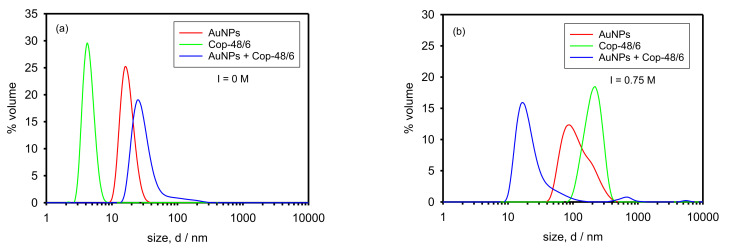
Size distribution by volume of the nanocomposite (AuNPs + Cop-48/6) at 25 °C. (**a**) Without added salt, and (**b**) with NaCl concentration 0.75 M. For an illustrative comparison, distributions of AuNPs and Cop-48/6 are also depicted.

For the sake of better comparison, Figure 5a represents in the same plot the size distribution functions by volume (coming from DLS measurements) of the AuNPs suspension, the Cop-48/6 solution, and the nanocomposite (AuNPs + Cop-48/6) resulting after mixing both systems at 25 °C and I = 0 M. As appreciated, the peak of the nanocomposite appears to slightly higher size than the peak of the AuNPs because copolymer-coated AuNPs are larger structures. Furthermore, some aggregates of Cop-48/6 with trapped AuNPs exist. On the other hand, most of the chains in the copolymer solution are forming random coils.

At I = 0.75 M (the previously established worst case for AuNPs aggregation) and 25 °C (Figure 5b), aggregation occurs both in the AuNPs suspension and in the Cop-48/6 solution. In these conditions, copolymer chains in solution are mostly forming aggregates or networks (no peak around 3 nm appears) although 25 °C is below the LCST. Both the high ionic strength that screens the copolymer charge and the high copolymer concentration favors the interchain association. However, the structures in the nanocomposite system (AuNPs + Cop-48/6) keep about the same size found at I = 0 M. On the one hand, it indicates that the copolymer prevents AuNPs aggregation either by forming core-shell structures or networks with trapped AuNPs. On the other hand, the copolymer chains do not appreciably aggregate because their charges are screened by the trapped AuNPs and the increase in the ionic strength has little effect on them. A few large structures also exist in the nanocomposite system which are evident in the distribution by intensity (not shown for simplicity) but, as it will be revealed by UV-vis spectroscopy, they are copolymer networks (always existing at any working condition) and not AuNPs aggregates.

**Figure 6:**
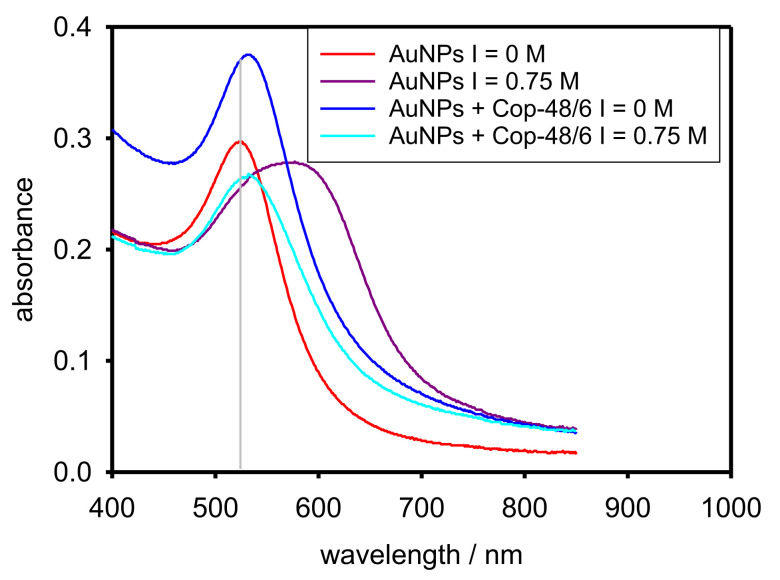
UV-vis spectra of the nanocomposite (AuNPs + Cop-48/6) at 25 °C in varying NaCl concentrations. For an illustrative comparison, UV-vis spectra of AuNPs are also depicted.

We can determine the aggregation of AuNPs in the nanocomposite by UV-vis spectroscopy since the plasmon peak is only due to the AuNPs and its position depends on their size. Figure 6 (update of Figure 7 in [15]) collects the UV-vis spectra coming from the AuNPs suspension and the nanocomposite both at I = 0 M and I = 0.75 M and 25 °C. It is observed that the plasmon peak appears approximately to the same wavelength in the original AuNPs suspension and in both nanocomposite systems (see solid grey line) indicating that AuNPs are about the same original size in the three systems. However, the plasmon peak is clearly red-shifted in the AuNPs suspension at I = 0.75 M indicating strong aggregation of AuNPs. Certainly, the plasmon peak in the nanocomposite systems appears slightly red-shifted, but that can be attributed to the proximity of the nanoparticles trapped in the copolymer networks. Thus, some of them are close enough in order to produce that slight shift [21] but preserve their individuality. On the other hand, the position of the plasmon peak in both nanocomposite systems is exactly the same indicating that no change in AuNPs size was provoked by salt addition. Therefore, AuNPs do not aggregate in the nanocomposite system.

**Figure 7:**
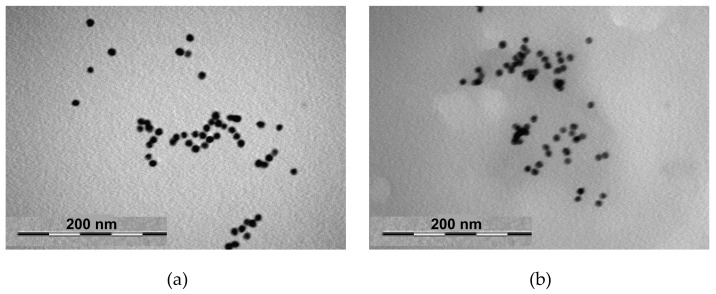
TEM images of the nanocomposite (AuNPs + Cop-48/6): (**a**) I = 0 M, (**b**) I = 0.75 M.

The above conclusions are confirmed by TEM images. Thus, Figure 7a (see also Figure 8 in [15]) corresponds to the nanocomposite at I = 0 M. It can be appreciated that some AuNPs are individually scattered through the solution. Those AuNPs must be coated by copolymer chains forming core-shell structures. On the other hand, also groups of AuNPs are observed which, although close to each other, preserve their individuality. Those AuNPs must be trapped in copolymer networks. Figure 7b (see also Figure 9 in [15]) corresponds to the nanocomposite system at I = 0.75 M. A picture similar to Figure 7a is observed: some AuNPs are scattered and some are grouped preserving their individuality. This is in agreement both with Figure 5, where the nanocomposite distribution is quite similar in Figure 5a,b and Figure 6 where the plasmon peak keeps its position. Some AuNPs seem closer in Figure 5b than in Figure 5a because at high ionic strength more compact copolymer networks are formed.

On the other hand, TEM images suggest that the trapped AuNPs are stuck on the surface of the Cop-48/6 networks, otherwise most of the images will show regions with very high AuNPs density and the individuality of the AuNPs could not be appreciated.

### 3.4. The Nanocomposite (AuNPs + Cop-48/6) in Varying Temperature

**Figure 8:**
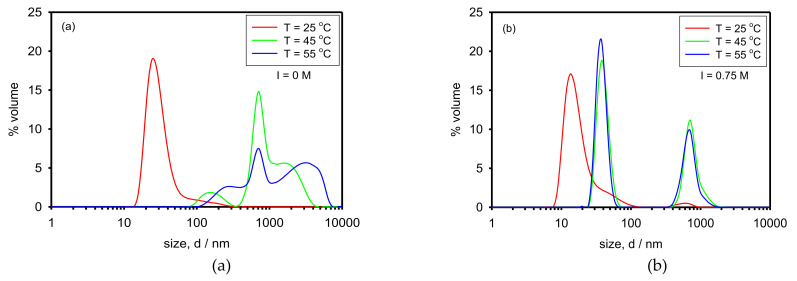
Size distribution by volume of the nanocomposite (AuNPs + Cop-48/6) at several temperatures. (**a**) Without added salt, and (**b**) with NaCl concentration 0.75 M.

We also studied the behavior of the nanocomposite (AuNPs + Cop-48/6) when temperature is increased, both at I = 0 M (Figure 8a) and I = 0.75 M (Figure 8b).

At 25 °C, below the LCST of the copolymer (42 °C), we obtain the same distributions depicted in Figure 5. In increasing the temperature above the LCST, peaks at large size values appear because the copolymer chains become hydrophobic and associate forming large networks.

**Figure 9:**
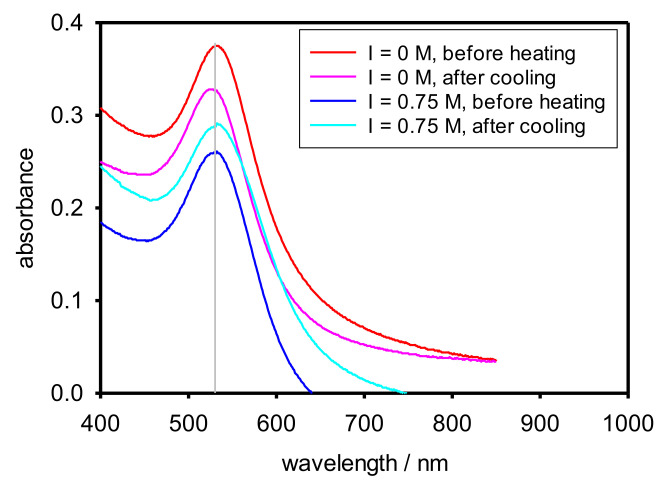
UV-vis spectra of the nanocomposite (AuNPs + Cop-48/6) at 25 °C in varying NaCl concentrations before heating and after cooling.

UV-Vis spectroscopy was used in order to elucidate if AuNPs aggregation occurred due to the heating process. Since we could only work at 25 °C, we measured the nanocomposite both before heating and after cooling. Figure 9 (that extends Figure 11 in [15]) clearly shows that the plasmon peak appears at the same wavelength for all of the cases. Therefore, it could be concluded that, regardless of the ionic strength, AuNPs do not aggregate during the process of increasing and decreasing the temperature.

## 4. Discussion and Conclusions

PNIPAAM_48_-b-PAMPTMA_6_(+), or Cop-48/6, is a positively charged amphiphilic copolymer with an LCST of about 42 °C that forms aggregates or networks by increasing the temperature and/or the ionic strength. At a temperature above the LCST, Cop-48/6 becomes hydrophobic due to the PNIPAAM block and interchain association occurs. On the other hand, the increase in ionic strength screens the charged PAMPTMA(+) block diminishing its hydrophilicity and favoring the interchain association.

When a Cop-48/6 solution is added to an AuNPs suspension, the electrostatic attraction between the AuNPs and the copolymer chains provokes the formation of core-shell structures and copolymer networks with individually trapped AuNPs. Thus, the expected AuNPs aggregation by increasing the ionic strength does not occur. AuNPs preserve also their individuality when the temperature is increased and large copolymer networks are formed. The AuNPs are scattered mainly on the surface of those networks in such a way that they are close enough but not in the necessary contact to form AuNPs aggregates what explains the slight red-shift of the plasmon peak. 

In sum, coating gold nanoparticles with the thermosensitive cationic diblock copolymer Cop-48/6 gives rise to a most peculiar aggregation behavior, which is modulated by temperature and ionic strength, and results in a nanocomposite in which individual AuNPs—rather than agglomerated—are dispersed in polymer networks which size varies strongly with the temperature. Thus, structures about 1000 nm are formed when the temperature exceeds the LCST. 

The size and polydispersity of the nanocomposite structure are not conducive to discovering an immediate application in the biomedical field, but the possibility of generating polymer networks containing small metallic nanoparticles scattered and immobilized on their surface regardless of the salinity and the temperature of the medium can have advantages in other fields like catalysis and nanoparticle cleaning. It must be considered that, in principle, several metallic nanoparticles other than gold (e.g., silver or different transition metals oxides) could also form this kind of nanocomposite. 

The role of small metallic nanoparticles (e.g., AuNPs) as catalytic materials is well recognized as well as the importance of the catalytic nanoparticle stabilization and support [22]. Furthermore, it is crucial for the catalyst recovery after its use. In this sense, the type of nanocomposite studied in this work is a good candidate for being used as a semi-heterogeneous catalyst and could be considered as a nanoreactor. Thus, Cop-48/6 networks are appropriate templates to anchored and stabilized high concentrations of small size nanoparticles that will keep their individuality independently of the medium conditions. Moreover, the size of the network structures could be tuned by varying the temperature. Both the effect of increasing the temperature and the network size can be positive for some chemical processes. Finally, due to its large size and molecular weight, the nanocomposite could be easily recovered by centrifugation. 

On the other hand, the current wide use of small metallic nanoparticles is a serious menace for environmental and human health [23]. Precisely, the small size and the spherical shape of many nanoparticles, which are crucial features for many of their applications, are also in part the origin of their toxicity [24]. A strategy to diminish this problem is designing nanoparticles with low toxicity [25], but that is not always possible and many small nanoparticles are released daily to the environment. Nowadays, there is an increasing concern about the environmental problems caused by the indiscriminate use of nanoparticles [23]. In this regard, the formation of nanocomposites as those studied in this work can be of interest in the search for a solution. Thus, nanoparticles keep their small size in those nanocomposites, as required for some applications like catalysis, but are tightly trapped in large structures, which could avoid or diminish the toxicity of the material (since it cannot enter living cells). Besides, the formation of big networks structures that trap nanoparticles can be used to collect dispersed nanoparticles and remove them from the medium by gravitational sedimentation or centrifugation, preferably before they are released to the environment. In this way, the formation of nanocomposites larger than 1000 nm can be used as a cleaning strategy for toxic nanoparticles.

## Data Availability

The data presented in this study are available upon request from the corresponding author.

## Supplementary Materials

The following are available online at https://www.mdpi.com/article/10.3390/polym13234066/s1, Figure S1: Equilibration time, Figure S2: Distribution by intensity vs. distribution by volume.
