# Supplementary material for: Aggregation of Gold Nanoparticles in Presence of the Thermoresponsive Cationic Diblock Copolymer PNIPAAM48-b-PAMPTMA6
Source: Polymers (Basel). 2021 Nov 23;13(23):4066. doi: 10.3390/polym13234066 (PMC8658865; doi:10.3390/polym13234066)
Supplement: Supplementary file 1 [file polymers-13-04066-s001.zip › polymers-1448294-supplementary.pdf]

Supplementary Materials

Aggregation of Gold Nanoparticles in Presence of the Thermoresponsive Cationic Diblock Copolymer $PNIPAAM_{48}$-b-$PAMPTMA_6$

David Herrera Robalino, María del Mar Durán del Amor, Carmen María Almagro Gómez and José Ginés Hernández Cifre *

Here, we show the plots that justify:

1) The choice of 10 min as equilibration time when heating the copolymer solution.
2) The choice of distribution by volume instead of distribution by intensity in order to identify the relevant structures existing in the copolymer solution.

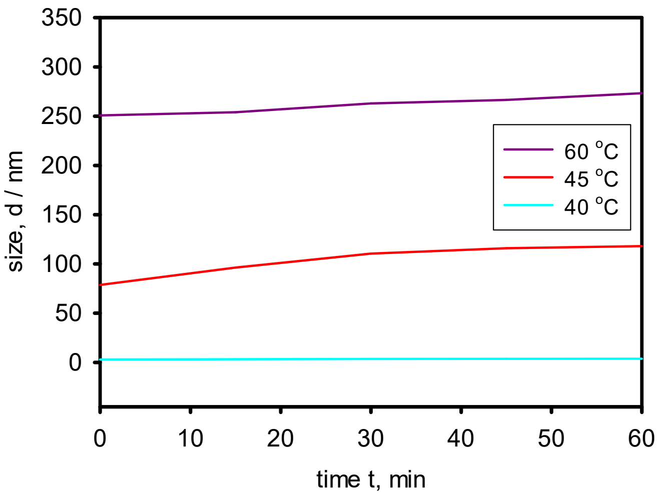

Figure S1. Equilibration time. Time evolution of the size of the Cop-48/6 in solution 0.01% w/w for several temperatures without added salt (I = 0 M).

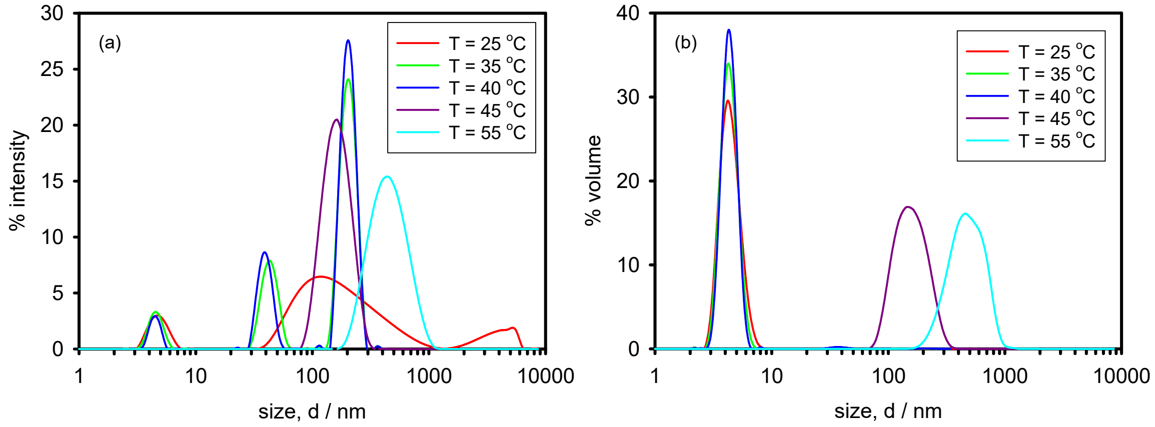

Figure S2. Distribution by intensity vs. distribution by volume. Size distributions of Cop-48/6 in solution 0.01% w/w in varying temperature and without added salt (I = 0 M): (a) by intensity (b) by volume.
